# Understanding and treating body image disturbances in eating disorders through body illusion interventions: a scoping review protocol

**DOI:** 10.1186/s13643-024-02458-8

**Published:** 2024-02-13

**Authors:** Sergio Navas-León, Ana Tajadura-Jiménez, Emma Motrico, Luis Morales, Mercedes Borda-Mas, Nerea Almeda, Milagrosa Sánchez-Martín

**Affiliations:** 1 Department of Psychology, Universidad Loyola Andalucía, Avda. de Las Universidades S/N, 41704 Dos Hermanas, Seville Spain; 2 UCL Interaction Centre, University College London, London, WC1E 6BT UK; 3 DEI Interactive Systems Group, Department of Computer Science, Universidad Carlos III de Madrid, 28911 Madrid, Spain; 4 Departamento de Personalidad, Evaluación y Tratamiento Psicológicos, Universidad de Sevilla, 41018 Seville, Spain

**Keywords:** Anorexia, Bodily illusions, Body image disturbance, Eating disorders, Mental health, Psychiatry, Public health, Scoping review

## Abstract

**Background:**

We plan a scoping review aimed to synthesize what is known about the use of sensory-driven body illusion (BI) interventions for understanding and treating body image disturbance (BID) in people diagnosed with clinical eating disorders (EDs) and people with subclinical ED symptomatology. Our study will provide an outline of the current literature, identify gaps within the literature, and suggest novel directions for future research.

**Methods/design:**

The scoping review process will be guided by the methodological framework of Arksey and O’Malley, subsequent recommendations by Levac et al., and the Preferred Reporting Items for Systematic Reviews and Meta-analysis Protocols Extension for Scoping Reviews guidelines. The following electronic databases will be systematically searched: MEDLINE (via PubMed), Web of Science, PsycINFO, and Scopus. Furthermore, to identify additional studies, we will use a search engine such as Google Scholar, and for grey literature, we will include Proquest for Dissertations and Theses. A search strategy has been identified and agreed upon by the research team in conjunction with a research librarian. Two researchers will screen the titles and abstracts independently and then assess the full text of the selected citations for the inclusion criteria. A third reviewer will be involved in cases of disagreement. Data will be extracted, collated, and charted to summarize all the relevant methods, outcomes, and key findings in the articles.

**Discussion:**

A better understanding of this topic will aid in the development and refinement of current treatments aimed at treating BID in people with EDs. Implications and recommendations for research, policy, and practice in the context of the ED community will be discussed.

**Systematic review registration:**

https://osf.io/3bcm6/?view_only=83b2e8a2445d4266909992e3dfb51929

**Supplementary Information:**

The online version contains supplementary material available at 10.1186/s13643-024-02458-8.

## Background

Eating disorders (EDs) are relatively common disorders among the general population (e.g. subthreshold EDs 2–3% of women in Europe) [1, 2]. Furthermore, they are associated with serious impairments in various life domains [3] and medical and psychopathological comorbidities [1], resulting in the highest mortality rates of all psychiatric illnesses [4]. EDs are collectively considered a serious health condition and thereby a public health issue. A core feature of EDs, specifically for anorexia nervosa (AN) and bulimia nervosa (BN), is the presence of body image disturbance (BID) [5].

BID commonly includes two components [6]: a perceptual component and an attitudinal component. The perceptual component refers to the inability to accurately estimate body size. The attitudinal component refers to negative feelings and thoughts towards the body. Traditionally, the latter has been seen as a critical component in current cognitive-behavioural therapy approaches [7], whereas the perceptual component has been overlooked [8]. One possible explanation is that the attitudinal component is handled first, with the hope that the perceptual component may subside in the later phases of the illness [9, 10]. Therefore, it remains “largely understudied” [11], with the current evidence-based practice being scarce [7, 12]. A large body of literature suggests that body size overestimation is common [13–16], persistent [17], and associated with poorer therapeutic outcomes [18]. Thus, body size overestimation is considered an independent feature that requires specialized treatment [8, 19]. It is therefore crucial to develop interventions that target the perceptual component [20]. Doing so may plausibly enhance the effectiveness of current cognitive-behavioural therapies [20].

Taking the lead on this issue, the relatively new area of multisensory integration could shed light on the mechanisms that underlie body size overestimation, opening new possibilities for its treatment. This ongoing research suggests that our mental body representations are continuously updated in response to the inputs that we receive from our body (i.e. visual, auditory, or haptic signals, among others) [21–23]. Experiments using sensory-driven body illusions have played a significant role in advancing this area by providing novel insights into the plasticity of our body representations. Body illusions are defined as any “psychological phenomenon in which the perception of one’s own body importantly deviates from the configuration of the physical one, e.g. in terms of size, location, or ownership” [24]. The experimental settings associated with these body illusions consist of exposing participants to multisensory conflicts [25]. As the prototypical paradigm, the rubber hand illusion (RHI) showed that it is possible to induce participants to perceive a rubber hand as a part of their body by touching the fake hand in synchrony with the participant’s hand (visuotactile integration) [26]. Derived from this original work, emerging literature is growing regarding body illusions. Prominent examples are ‘the full-body illusion’ [27], ‘the body-swap illusion’ [28], or ‘the Pinocchio illusion’ [29], among others (see [24, 30] for more details). Thus, given the evident malleability of our bodily representations, some authors have suggested that these body illusions could have clinical implications in terms of understanding and treating the perceptual component [31].

Following this promising hypothesis, underpinning research regarding body illusions and their application in the ED field is on the rise. Overall, the ongoing evidence relies on the RHI paradigm [17, 32–36]. Nevertheless, other studies have applied the full-body illusion [37–39], the size-weight illusion [40], or even the sound-induced flash illusion [41] to populations with clinical or subclinical EDs. In summary, these experimental studies report that participants diagnosed with clinical EDs and participants with subclinical ED symptomatology compared to healthy controls are prone to show a poorer integration of diverse sensory inputs (e.g. integration of visual and proprioceptive information or visual and auditory information). Derived from these findings, some authors have speculated a bias towards processing information on a visual basis [37]. For example, Keizer et al. [37] used the full-body illusion in patients with AN after blocking the visual input of their own body and reported that reducing the overestimation of body size is possible, with results lasting at least 2 h and 45 min after the illusion was induced. Therefore, given that those experimental settings surrounding body illusions can be used to “reactivate and correct” other sensory modalities [42], it has been suggested that future interventions should manipulate exteroceptive or interoceptive bodily signals in an attempt to reduce body size overestimation [37].

### Study rationale/objective

Sensory-driven body illusions, as previously indicated, are considered potential experimental paradigms for understanding and treating BIDs in EDs. Nonetheless, to date, only two review studies have evaluated the effectiveness of body illusions in the ED field [25, 43]. Matamala et al. (2020) aimed to discuss different body illusions for changing body representation in both healthy and clinical populations, presenting some mental illness conditions. Those conditions involved obsessive–compulsive disorders, schizophrenia, EDs, bipolar disorders, post-traumatic stress disorders, depression, and autism. Among the results, they analysed nine studies related to the use of body illusion in EDs, summarizing the main findings and outlining the potential clinical utility of this novel line of research. However, given the wide heterogeneity of objectives, methodological research practices, and clinical outcomes of the studies in question, a deeper examination of these studies is needed. Turbyne et al. (2021) carried out a systematic review and a meta-analysis aimed at elucidating the effectiveness of the full-body illusion, which uses virtual reality (exposing virtual bodies or body parts varying in size and/or shape) to reduce BID in both clinical and nonclinical populations. They found 12 studies, and only six consisted of ED samples. For this population, they confirmed the effectiveness of using virtual reality-generated full-body illusions to influence the perception or attitude towards the patient’s own body. However, only one type of body illusion has been investigated despite the wide variety of body illusions tested on ED samples to date.

Given the lack of research in this body of literature, to the best of our knowledge, no further attempts following a scoping review (hereafter, ScR) approach have been made that target all body illusions applied to participants diagnosed with clinical EDs and participants with subclinical ED symptomatology. Thus, we plan an ScR aimed at synthesizing what is known about body illusions for understanding and treating BIDs in this population.

## Methods/design

### Protocol design

Due to the diversity of the literature, an ScR will be employed. Specifically, this framework allows us to synthesize the literature and to explore, map, and understand the extent of the currently available literature [44, 45]. Furthermore, it allows us to identify and summarize knowledge gaps, which will assist with making recommendations for future research [44, 45]. The current protocol and future corresponding ScR will follow Arksey and O’Malley’s five-stage methodological framework: (1) identifying the research question; (2) identifying the relevant studies; (3) selecting studies; (4) charting the data; and (5) collating, summarizing, and reporting the results. The optional sixth stage, ‘consultation with relevant stakeholders’, will be included as part of this review to identify additional resources. In addition, we will follow the subsequent refinement and recommendations of these steps outlined by Levac et al.’s [45] guidelines as well as Colquhoun et al.’s [46] updated approach to conducting ScRs. We will pay particular attention to 2020 recommendations from the Joanna Briggs Institute (JBI) [47]. The Preferred Reporting Items for Systematic Reviews and Meta-Analyses Extension for ScRs (PRISMA-ScR) [48] (see Additional supplemental file 1) will be used to report outcomes of the final ScR. The consideration of these frameworks and recommendations will ensure clear methodological and transparent processes that can be replicated. The ScR records and data will be preserved on a computer and a hard drive for backup purposes. To secure the data, the computer files will also be linked to the campus cloud. At present, ScR protocols are ineligible for registration in the PROSPERO database [47]. Thus, the protocol was registered in the Open Science Framework [49].

### Research team

The review will be conducted by a team of experts in the field of EDs (MBM, NAM, Ph.D), multisensory integration (ATJ, Ph.D), experimental psychology (LM, Ph.D), and methodology (MSM, EM, Ph.D), along with a research assistant (SNL).

### Changes to the protocol

Each stage may be revised further as the review advances, following the recommended iterative methodology for ScRs [44, 45]. The final report shall explicitly document any procedure deviations or revisions, as well as the reasons for these modifications as advocated by the JBI [50]. The deviations from this protocol will be tracked on the ScR’s Open Science Framework project.

### Stage 1: Identifying the research question

The primary research question (RQ) of this ScR is as follows: What is currently known about sensory-driven body illusions applied with participants diagnosed with clinical EDs and participants with subclinical ED symptomatology? In addition, during the preliminary exploratory review, we identified the following secondary RQs:

- RQ1) What are the aims of these studies (e.g. investigating multisensory integration basis and/or developing novel evidence-based interventions, specific BIs studied)?
- RQ2) What are the research methods involved in these studies?
- RQ3) What are the major findings found in these studies?

The objective of the study was formulated according to the above research questions. Nevertheless, the identification of additional research questions may be possible due to the generation of questions as an iterative process, informed by emerging themes that appear as the review team becomes increasingly familiar with the body of literature.

### Stage 2: Identifying relevant studies

#### Eligibility criteria

To identify relevant studies, according to Peters et al. [47], a ScR question should include elements of the PCC mnemonic (population, concept, and context) and will also inform inclusion and exclusion criteria and, consequently, the literature search strategy. This framework is more appropriate for ScRs than the ‘population, intervention, comparator and outcome’ (PICO) framework commonly used for systematic reviews, as it allows for the consideration of publications that may not feature all four PICO elements (e.g. lacking an outcome or comparator/control). Therefore, we will base our search strategy on the PCC framework described in Table 1. The suitability of the draft inclusion and exclusion criteria will be iteratively determined. At the onset of the ScR process, the authors and an expert librarian will discuss the exclusion and inclusion criteria. At this stage, all members of the research team will be satisfied that the search strategy was appropriate for the actual review.

**Table 1:** Eligibility criteria, based on study population, concept, context, and types of evidence

	**Inclusion**	**Exclusion**
**Population**	Participants of any age range with any of the following conditions: a an ED meeting DSM or other clinical criteria; b presence of subclinical ED (determined by questionnaire cut-off, clinical interviews or clinical judgements)	Other populations not meeting the inclusion criteria
**Concept**	Describe the implementation of body illusions.	Studies not considering body illusions.
**Context**	Any context. The context will not be limited to any specific geographic location	
**Types of evidence sources**^a^	- Peer-reviewed studies will be considered since they provide a high standard of evidence and reliability -Grey literature will include academic theses/dissertations, primarily to identify references to relevant peer-reviewed articles.	• Qualitative study designs such as grounded theory, ethnography, phenomenology, and action research and qualitative descriptive design. In addition, commentaries, opinion pieces, letters, editorials, trial registrations, evaluation reports, abstracts, books, book chapters, or book reviews • Systematic and literature reviews or protocols will not be included, but will instead be used to identify potentially relevant studies, depending on the research question • Conference proceedings due to limited detail in their abstracts • Articles for which we cannot obtain the full text
**Design**	Observational, analytical, cross-sectional, and longitudinal studies will be considered. The article must include statistical analyses that place participants diagnosed with clinical EDs and/or participants with subclinical ED symptomatology and healthy participants into separate groups	
**Language**^a^	Studies from any language	
**Time frame**^a^	Studies from any year, given the desired breadth of the review	

^a^Not part of the PCC framework. Added by the authors to highlight included and excluded evidence types

### Sources of information

The research team, in collaboration with an expert university librarian, will undertake a comprehensive search of the literature within the following primary databases: MEDLINE (via PubMed), Web of Science (WOS), PsycINFO, and Scopus. These databases were chosen to capture a comprehensive sample of the published literature. It should be noted that MEDLINE, Scopus, and WOS have been recommended for adequate and efficient search coverage [51, 52]. Likewise, PsycINFO, as the world’s largest resource, devoted to peer-reviewed literature in behavioural science and mental health [53]. Nevertheless, to identify additional studies not found through the primary database searches, a literature search will be conducted on bibliographical search engines such as Google Scholar. Following prior recommendations [52], we will use the first 200 references, sorted by relevance and then by time. If the number of references from other databases is low, the number of references from Google Scholar will be limited to 100 [52]. To ensure that some grey literature is included, we will include ProQuest Dissertations & Theses, from which only the first 100 items (ordered by relevance) will be reviewed because further screening is unlikely to provide additional relevant material [54]. In addition, due to the nature of the topic, we do not expect any grey literature in the form of policy documents and governmental and organizational reports.

### Search strategy

The search will be conducted according to the three steps of the JBI methodology for ScRs [50]:

1. The first step involved a limited preliminary search of PubMed in September 2021. This initial search sought to identify articles using body illusion interventions in EDs and was followed by analysis of the text words contained in the title and abstract and of the index terms used (Medical Subject Headings, MeSH). Boolean operators (AND/OR) were used to create search strings that combined the information collected. Table 2 provides a sample of the preliminary search string. A PubMed pilot sample search according to the PCC approach is shown in additional supplemental file 2.
2. The second step involves identifying additional studies not found through the database searches. The bibliographical references of included papers about the use of body illusions in EDs will be checked to capture any papers potentially missed in the electronic databases (snowball searching). Furthermore, citation tracking analysis through Google Scholar or other search databases for the same purpose will be performed on the included body illusions studies of the databases if available. If warranted, the authors of the included articles will be contacted for further information.
3. Finally, to ensure that key articles are captured, the final search strategy will be designed collectively and iteratively with the help of an expert librarian, who will provide suggestions and verifications regarding the appropriate search string and adaptation of search strategies across the different sources of information (always following PCC format) and input from the research team.

**Table 2:** Search strategy for MEDLINE

**Step**	**Keywords searched**
Concept	body illusion*[Title/Abstract] OR bodily illusion*[Title/Abstract] OR embodiment[Title/Abstract] OR multisensory[Title/Abstract] OR illusions[MeSH Major Topic]
Population	anorexia nervosa [Title/Abstract] OR anorexic[Title/Abstract] OR bulimia[Title/Abstract] OR bulimic[Title/Abstract] OR eating disorder*[Title/Abstract] OR disordered eating [Title/Abstract] OR “obese” [Title/Abstract] OR “overweight” [Title/Abstract] OR “obesity” [Title/Abstract] OR Feeding and Eating Disorders[MeSH Major Topic] OR Obesity [MeSH Major Topic)
Context	No restrictions

The full search strategy will be included in an additional supplemental file detailing the database, search string, the number of articles retrieved and date.

### Stage 3. Selecting the studies

After the research strategy is identified, all identified citations will be collated and uploaded into Mendeley. The remaining citations will then be exported to a WEB-based platform designed for data management that serves as a screening tool [55] through which duplicates will be removed. Before commencing the selection process, two independent reviewers (R1 and R2) will conduct a calibration exercise, in duplicate, to ensure reliability in correctly screening for inclusion. To accomplish this, reviewers will check a randomly selected sample of the collected citations (i.e., 10%). Interrater agreement will be calculated with Cohen’s Kappa statistics (κ). We will consider an interrater reliability κ ≥ 0.8 a high level of agreement [56]. Therefore, if low agreement is observed, eligibility criteria will be adjusted until an acceptable κ value is reached. The overall kappa will be reported. Once this phase is completed, we will conduct a two-phase screening process to assess the relevance of studies identified in the search, which will be continuously monitored guided by the eligibility criteria described above. First, the same pair of reviewers (R1 and R2) will examine titles and abstracts in duplicate. They will meet at the beginning, midpoint, and final stages of the title and abstract screening process to discuss any problems or ambiguities related to study selection. The agreement of two reviewers will be required to exclude the citations collected. If there is any doubt (e.g., Yes/No regarding study’s target population), the article will not be deleted until it has been thoroughly evaluated in Step 2. Second, the full text of the selected citations will be assessed by the same pair of reviewers (R1 and R2) in detail against the inclusion criteria. A request to the corresponding author or an interlibrary loan through our local library system will be made to receive the full document. Any disagreements that arise among the reviewers at each two-stage of the study selection process will be resolved through discussion. Nevertheless, if there are any discrepancies regarding which articles to include or exclude and/or the reasons for this decision, a third senior reviewer (R3) will be consulted to make the final decision. When the same data are reported in more than one publication (e.g. in a journal article and electronic report), only the article reporting the most complete dataset will be used. Reasons for exclusion will be provided in an additional supplemental file in the final review report. Additionally, the study selection process will be documented in a PRISMA flowchart [57] (see Additional supplemental file 3), permitting replication and comparison of any further studies.

### Stage 4. Charting the data

Following guidelines from the JBI Reviewer’s Manual [50], a bespoke a priori tabular chart, organized into a spreadsheet via Microsoft Excel 365 software, will be created for data extraction from the included studies. Table 3 outlines the standard bibliographic information.

**Table 3:** Sample data charting form

a) Authors
b) Year of publication
c) Origin/country in which study was conducted
d) Study design
e) Aims/scope
f) Sampling strategy
g) Study population
h) Sample size
i) Methodology
j) Intervention exposure type (if applicable) and comparison group (if applicable)
k) Duration of the exposure/intervention (if applicable)
l) Duration of the exposure/intervention (if applicable)
m) Outcomes assessment and method to assess associations (if applicable)
n) Key findings that relate to the ScR question

Extracted from JBI Reviewer’s Manual, 11.2.7 Data extraction [49]. This will be further refined as the review progresses

Initially, to answer the research question and study purpose, the research team will meet to decide which variables to extract. Thereafter, to ensure accurate and reliable data collection, the data charting form will be piloted to test its applicability by using a random sample of three relevant studies identified from database searches. This will be performed independently by two reviewers (R1 and R2) and will not be blinded to the authors of the study/document. If poor agreement is found (e.g., text discrepancy or missing information), the tabular chart form will be revised iteratively, and the training exercise will be repeated to increase interrater reliability. For transparency in the reporting, any modifications will be detailed in the final ScR. Thereafter, once the accuracy and comprehensiveness of the tabular chart has been confirmed, we will proceed to full data extraction. This process will be completed by one researcher (R1) and verified by a second researcher (R2) with regular meetings with the research team throughout the process to discuss any iterative changes to the data charting fields until consensus. Therefore, the tabular chart form will be continually revised to elicit whether further information is required or if fields are not relevant and should be deleted. Additionally, it is expected that any relevant data not gathered during the initial data extraction step will be added to the chart iteratively through adjustments. If an article contains unclear or missing data, we will contact the relevant author via email for clarification or further information. Once extracted, all data will be compiled into a spreadsheet via Microsoft Excel 2016. In the final report, the tabular chart titles will be given in a summary table, and all charted data will be made publicly available.

The risk of bias will be assessed with the Risk Of Bias In Non-randomized Studies of Interventions (ROBINS-I) Tool [58] under the following 7 domains of bias: confounding, selection of participants, classification of interventions, deviation from protocol, missing data, measurement of outcomes, and selection of the reported result. A recent review in the field found ROBINS-I to be the preferentially recommended tool in estimating the comparative effectiveness of interventions in medical studies not adopting randomization [59]. The aim of the risk of bias is merely descriptive rather than selective and will aid in data analysis interpretation. Thus, all studies will remain included. Two reviewers (R1 and R2) will use the checklist tool and independently assess the risk of bias. A kappa coefficient will be obtained. Any disagreements will be discussed and resolved by consensus.

### Stage 5. Collating, summarizing, and reporting the results

The tabular results will be supplemented by a narrative overview mapping the findings from the extracted data (Stage 4). We will summarize the gathered data through a deductive analytical approach and discuss how the findings connect to the study question and objectives. Charted data will be synthesized quantitatively and qualitatively. Both approaches will be conducted by one reviewer (R1) as an iterative process in consultation with the review team. Quantitatively, summary statistics will be used to describe the current volume, yearly distribution, countries of origin, sample characteristics, and methodological design. Additionally, clusters and heatmaps of frequently occurring terms in the included studies will be visualized with VOSviewer [60]. Qualitatively, findings will be organized into thematic categories such as aims, methodological design, key findings, and gaps in the literature, among others. To minimize bias and ensure a consistent approach in reporting the results, the research team will meet to discuss the thematic categories. If necessary, additional headings will be used to summarize the studies if findings are not sufficiently communicated through the aforementioned taxonomy. As necessary, tables and diagrams will be utilized to illustrate findings augmented by narrative text according to key findings and knowledge gaps. Moreover, the strengths and limitations of the ScR will be discussed alongside future recommendations for research. We will consider the meaning of the results in terms of the broader implications for research, policy, and practice in the context of the ED community.

### Stage 6. Consulting with stakeholders

As suggested by Levac et al. [45] we will include consultation with stakeholders. Hence, we will address EDs and multisensory integration experts by purposeful snowball sampling, when preliminary results from Stage 3 are available, to identify additional relevant literature to include in the review. The consultation exercise will be conducted through an electronic mailing list.

### Ethics and dissemination

The ScR will collect and examine data from literature and therefore does not require prior ethical approval. Our dissemination strategy will include a submission for publication in a peer-reviewed journal and presentations at academic conferences or symposia. More locally, the findings of the review will be disseminated through Universidad Loyola Andalucía, Universidad Carlos III de Madrid, University College of London and social media accounts by members of the research team and shared with ED organizations by sending an email with a user-friendly evidence summary and a copy of the peer-reviewed article.

## Discussion

To the best of our knowledge, this is the first ScR to identify the scope of evidence and gaps in the literature regarding body illusions applied to EDs. From the knowledge obtained in this review, we anticipate that the findings of this ScR will guide and inspire research into the treatment of EDs, where BIDs are considered a core symptom. We consider that more research is needed to lay the groundwork for future BID interventions employing body illusions. A better understanding of this topic will aid in the development and refinement of current treatments aimed at treating BIDs in people with EDs. Implications for future public policies seeking to prevent and treat EDs will be discussed. Also, inherent risks and ethical considerations associated with their misuse will be discussed. To this end, the proposed ScR will address the following objectives: (a) to provide a comprehensive overview of the current literature and (b) to identify and analyse knowledge gaps to guide future research strategies and lines of action.

### Supplementary Information

**Additional file 1.** Preferred Reporting Items for Systematic reviews and Meta-Analyses extension for Scoping Reviews (PRISMA-ScR) Checklist.

**Additional file 2. **Preliminary search on PubMed.

**Additional file 3.** PRISMA 2020 flow diagram for new systematic reviews which included searches of databases, registers and other sources.

## Data Availability

The datasets generated and/or analysed during the current study will be available in the Open Science Framework.
