# Supplementary material for: Understanding and treating body image disturbances in eating disorders through body illusion interventions: a scoping review protocol
Source: Syst Rev. 2024 Feb 13;13:65. doi: 10.1186/s13643-024-02458-8 (PMC10863300; doi:10.1186/s13643-024-02458-8)
Supplement: Supplementary file 2 — Additional file 2. Preliminary search on PubMed. [file 13643_2024_2458_MOESM2_ESM.pdf]

15/06/22

Search number	Search terms/combinations	Number of ítems found
#1	"body illusion*"[Title/Abstract] OR "bodily illusion*"[Title/Abstract] OR "embodiment"[Title/Abstract] OR "multisensory"[Title/Abstract] OR "illusions"[MeSH Major Topic]	16.838
#2	anorexia[Title/Abstract] OR anorexic[Title/Abstract] OR bulimia[Title/Abstract] OR bulimic[Title/Abstract] OR eating disorder*[Title/Abstract] OR disordered eating [Title/Abstract] OR “obese” [Title/Abstract] OR “overweight” [Title/Abstract] OR “obesity” [Title/Abstract] OR Feeding and Eating Disorders[MeSH Major Topic] OR Obesity [MeSH Major Topic)	449.783
#3	#1 AND #2	179
