# Supplementary material for: Understanding and treating body image disturbances in eating disorders through body illusion interventions: a scoping review protocol
Source: Syst Rev. 2024 Feb 13;13:65. doi: 10.1186/s13643-024-02458-8 (PMC10863300; doi:10.1186/s13643-024-02458-8)
Supplement: Supplementary file 3 — Additional file 3. PRISMA 2020 flow diagram for new systematic reviews which included searches of databases, registers and other sources. [file 13643_2024_2458_MOESM3_ESM.pdf]

PRISMA 2020 flow diagram for new systematic reviews which included searches of databases, registers and other sources

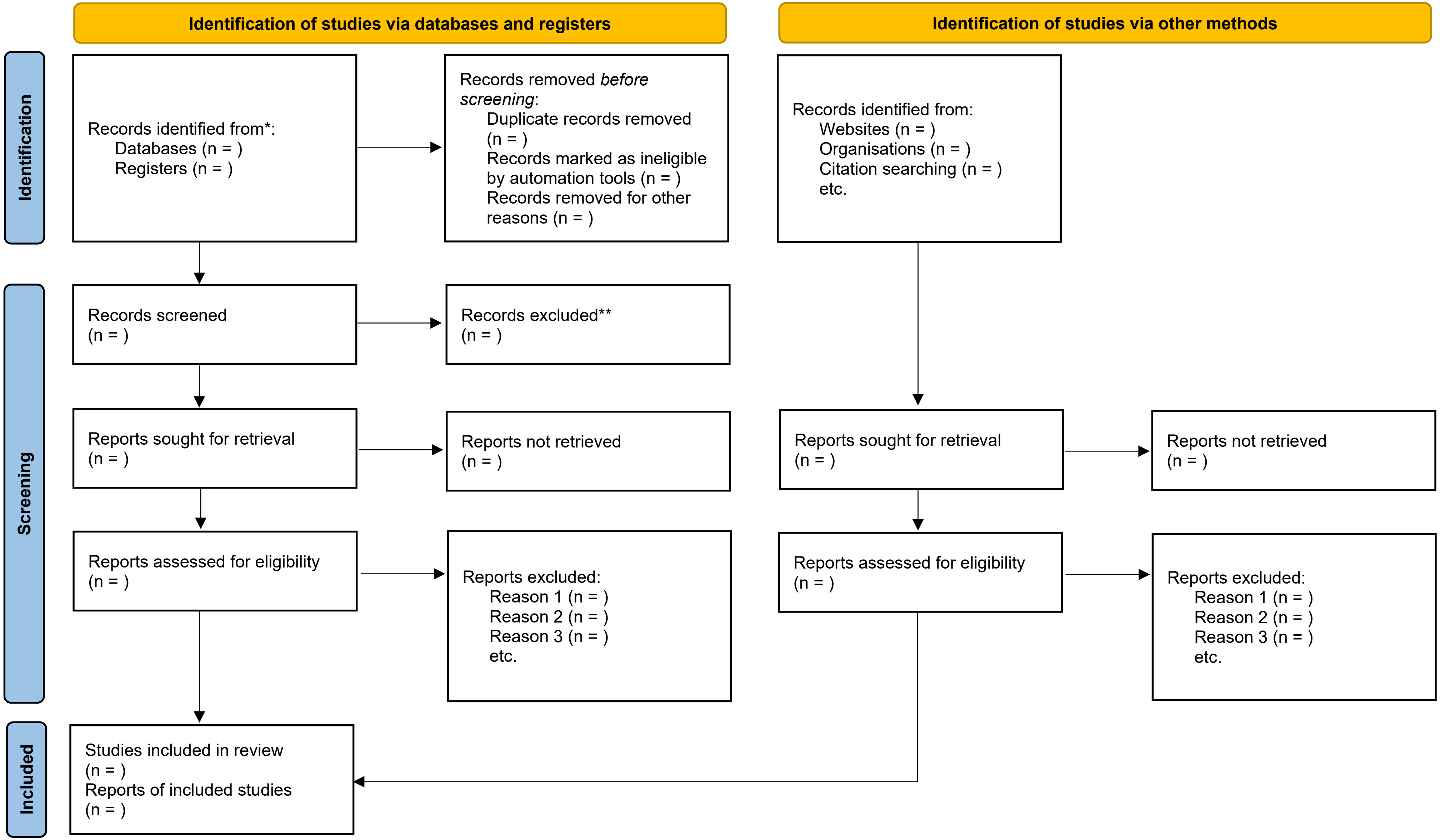

*Consider, if feasible to do so, reporting the number of records identified from each database or register searched (rather than the total number across all databases/registers).
**If automation tools were used, indicate how many records were excluded by a human and how many were excluded by automation tools.

From: Page MJ, McKenzie JE, Bossuyt PM, Boutron I, Hoffmann TC, Mulrow CD, et al. The PRISMA 2020 statement: an updated guideline for reporting systematic reviews. BMJ 2021;372:n71. doi: 10.1136/bmj.n71. For more information, visit: http://www.prisma-statement.org/
